# Euglycemic Diabetic Ketoacidosis Treatment Protocol With Increased Dextrose Supplementation to Prevent Hypoglycemia

**DOI:** 10.1111/acem.70278

**Published:** 2026-03-27

**Authors:** Alexander T. Clark, Jordan Sell, Lynn Ang, Nazanene H. Esfandiari, James A. Cranford, Nathan L. Haas

**Affiliations:** ^1^ Division of Critical Care, Department of Emergency Medicine University of Michigan Ann Arbor Michigan USA; ^2^ Department of Emergency Medicine University of Michigan Ann Arbor Michigan USA; ^3^ Division of Metabolism, Endocrinology, and Diabetes, Department of Internal Medicine University of Michigan Ann Arbor Michigan USA

**Keywords:** dextrose, euglycemic diabetic ketoacidosis, hypoglycemia, insulin, intravenous fluids

Euglycemic diabetic ketoacidosis (eDKA) consists of ketosis and elevated anion gap metabolic acidosis without hyperglycemia, and represents 10% of cases of diabetic ketoacidosis (DKA) [1]. Similar to hyperglycemic DKA, eDKA is triggered by physiologic stress and insulin deficiency leading to lipolysis and ketogenesis, but without hyperglycemia due to mechanisms such as exogenous insulin use, reduced food intake, liver disease, sodium‐glucose co‐transporter 2 inhibitor (SGLT2i) therapy, pregnancy, or impaired gluconeogenesis from alcohol use [2]. eDKA must be distinguished from other causes of ketosis and elevated anion gap metabolic acidosis, including alcoholic ketoacidosis and starvation ketoacidosis, based on careful history, physical examination, and review of medications, as the treatment of each condition differs [3]. The absence of hyperglycemia in eDKA poses a therapeutic challenge, as insulin needed for ketoacidosis clearance carries a risk of iatrogenic hypoglycemia [4]. Our previous work comparing 44 patients with eDKA to 585 with hyperglycemic DKA managed with the same protocolized fixed‐dose intravenous insulin infusion and dextrose supplementation observed over three times the incidence of hypoglycemia during treatment of eDKA compared to hyperglycemic DKA (18.2% vs. 4.8%, *p* = 0.02) [5]. This difference was noted despite the eDKA cohort having 6 h shorter total duration of IV insulin infusion. Albert et al. [6] similarly observed a standard hyperglycemic DKA protocol (lower dose titratable insulin infusion) for treatment of eDKA paradoxically prolonged time to eDKA resolution, highlighting the need for eDKA‐specific treatment strategies. To facilitate adequate insulin doses needed to correct ketoacidosis while mitigating risks of treatment‐related hypoglycemia, greater dextrose supplementation during eDKA treatment has been proposed [7, 8]. While this strategy is extrapolated from physiologic reasoning and supported by expert opinion, outcome data of eDKA‐specific treatment strategies are lacking. The objective of this study was to compare key safety and resource utilization outcomes before and after implementation of an eDKA‐specific treatment strategy of higher dextrose supplementation.

We conducted a retrospective observational analysis of all emergency department (ED) patients with eDKA treated using a DKA order set at an academic medical center in the United States. The Institutional Review Board at the University of Michigan reviewed and approved this study. In May 2023, our institution implemented an eDKA‐specific treatment protocol consisting of increased supplemental dextrose with fixed rate insulin. The *pre‐intervention* cohort included patients with eDKA treated with the standard hyperglycemic DKA protocol between August 2015 and May 2023, and the *post‐intervention* cohort included patients with eDKA treated with the eDKA‐specific protocol from May 2023 through November 2024. Inclusion criteria were ED patients ≥ 18 years old with eDKA on initial laboratories (pH ≤ 7.30, bicarbonate ≤ 18 mmol/L, anion gap ≥ 10, blood glucose level ≤ 250 mg/dL [9]). Exclusion criteria were hyperglycemic DKA (initial glucose > 250 mg/dL) and treatment with more than one insulin infusion order set.

Both cohorts received fixed‐rate intravenous insulin infusion (0.1 units/kg/h), similar fluid (250 mL/h) and protocolized electrolyte delivery, but different titratable dextrose supplementation in response to hourly glucose checks. The methodology [10] used in the *pre‐intervention* cohort standardized for blood glucose:

- > 250 mg/dL: non‐dextrose‐containing fluids
- 150–250 mg/dL: 5% dextrose‐containing fluids
- < 150 mg/dL: 10% dextrose‐containing fluids

In comparison, the *post‐intervention* cohort (eDKA‐specific protocol) standardized for blood glucose:

- ≥ 250 mg/dL: 5% dextrose‐containing fluids
- < 250 mg/dL: 10% dextrose‐containing fluids

Accordingly, patients in the *post‐intervention* cohort (eDKA‐specific protocol) received a higher rate of dextrose supplementation than the *pre‐intervention* cohort. Guidelines in both groups recommended intravenous insulin until resolution of DKA, defined as pH > 7.30, bicarbonate > 15 mmol/L, glucose < 200 mg/dL, anion gap < 12, and tolerating diet [9]. At that point, subcutaneous insulin was recommended with 2 h of intravenous insulin infusion overlap prior to discontinuation.

Data were extracted from the electronic health record (EHR) using an automated query. Demographics included age, sex, and weight. The primary outcome was incidence of hypoglycemia (< 70 mg/dL) or clinically significant hypoglycemia (< 54 mg/dL) while on intravenous insulin infusion [11]. Additional safety outcomes included incidence of hypokalemia, defined as mild < 3.3 mmol/L, moderate < 3.0 mmol/L, and severe < 2.7 mmol/L. Resource utilization outcomes included duration of insulin infusion (interval from infusion start time to last infusion stop time), time from ED presentation to first bicarbonate > 18 mmol/L, time from ED presentation to first long acting subcutaneous insulin administration, initial admission to non‐intensive care unit (ICU) with transfer to ICU within 24 h, total hospital charges, length of stay (LOS), and in‐hospital mortality. Comparisons between pre‐intervention and post‐intervention cohorts used independent‐groups *t*‐tests for continuous variables and chi‐squared tests for binary variables. An alpha level of 0.05 was used for all analyses, all hypothesis tests were two‐sided, unstandardized effect sizes (*d*) and 95% confidence intervals were estimated, and *p* values for all test statistics were calculated based on cluster‐robust standard errors adjusted for multiple visits clustered within patients. Analyses were conducted with the Stata (Release 15.1, Stata Corp., 2017) software package.

We identified 44 encounters from 38 patients with eDKA in the pre‐intervention cohort treated with the standard hyperglycemic DKA protocol, and 15 encounters from 14 patients in the post‐intervention cohort treated with the eDKA‐specific protocol (Table 1). Patients in the post‐intervention cohort were older (51.7 vs. 31.1 years, *p* < 0.001). There were no statistically significant differences in presenting laboratory values including blood glucose (162 vs. 195 mg/dL, *p* = 0.07), pH, bicarbonate, anion gap, or potassium. Incidence of hypoglycemia (glucose < 70 mg/dL) while on intravenous insulin was 13.3% in the post‐intervention cohort vs. 18.2% in the pre‐intervention cohort (*d*[95% CI] 4.9 [−16, 25.7]; *p* = 0.65). The incidence of clinically significant hypoglycemia (< 54 mg/dL) was similar (6.7% vs. 4.5%, *d*[95% CI] 2.1 (−25.7, 16.0); *p* = 0.77). There were no statistically significant differences in incidence of mild, moderate, or severe hypokalemia, and no patients died. The post‐intervention cohort was treated with IV insulin infusion for longer (26.7 h vs. 13.5 h, *d*[95% CI] 13.3 (9.9, 17.1); *p* = 0.04). There were no statistically significant differences in time to the first bicarbonate level greater than 18 mmol/L, total hospital charges, or total hospital LOS.

**TABLE 1:** Presenting demographic and laboratory characteristics of eDKA patients presenting to the ED in this study and stratified into pre‐ and post‐intervention cohorts.

	Pre‐intervention (*n* = 44)^a^: ED patients with eDKA treated with standard DKA protocol	Post‐intervention (*n* = 15)^b^: ED patients with eDKA treated with eDKA‐specific protocol with increased dextrose supplementation	*d* (95% CI)	*p*
*Demographics*				
Mean age, years (95% CI)	31.1 (27.1, 35.1)	51.7 (43.4, 60.0)	−20.6 (−30.0, −11.2)	< 0.001
Female sex, *n* (%)	30 (68.2)	9 (60.0)	8.2 (21.9, 38.2)	0.59
Mean weight, kg (95% CI)	70.2 (63.5, 76.9)	75.2 (63.1, 87.3)	−5.2 (−19.1, 5.0)	0.48
Mean presenting laboratory values (95% CI)				
pH	7.17 (7.14, 7.19)	7.21 (7.16, 7.26)	−0.04 (−0.10, 0.02)	0.17
Bicarbonate (mmol/L)	11.9 (10.7, 13.0)	11.9 (10.0, 13.7)	0.0 (−2.3, 2.3)	1
Anion gap	21.6 (19.8, 23.4)	22.5 (19.4, 25.6)	−0.9 (−4.6, 2.7)	0.61
Glucose (mg/dL)	195 (182, 208)	162 (130, 194)	33 (3, 68)	0.07
Potassium (mmol/L)	4.3 (4.1, 4.6)	4.6 (4.1, 5.2)	−0.3 (−0.9, 0.3)	0.31
*Safety outcomes*				
Hypoglycemia incidence, *n* (%)				
Glucose < 70 mg/dL	8 (18.2)	2 (13.3)	4.9 (−16.0, 25.7)	0.65
Glucose < 54 mg/dL	2 (4.5)	1 (6.7)	−2.1 (−25.7, 16.0)	0.77
Hypokalemia incidence, *n* (%)				
Potassium < 3.3 mmol/L	12 (27.3)	4 (26.7)	0.6 (−2.6, 2.7)	0.96
Potassium < 3.0 mmol/L	3 (6.8)	2 (13.3)	−6.5 (−25.6, 12.6)	0.5
Potassium < 2.7 mmol/L	1 (2.3)	0 (0.0)	2.3 (−6.7, 2.5)	0.32
Admission to non‐ICU with transfer to ICU within 24 h, *n* (%)	0	0		
Hospital mortality, *n* (%)	0	0		
*Resource utilization outcomes*				
Mean time on insulin infusion, hours (95% CI)	13.5 (10.1, 16.9)	26.7 (14.5, 39.0)	−13.3 (−17.1, −9.9)	0.04
Mean hours to first bicarbonate > 18 mmol/L, (95% CI)	12.3 (10.6, 14.1)	14.3 (10.8, 17.7)	−1.9 (−5.9, 2.0)	0.32
Mean time to first long‐acting subcutaneous insulin administration, hours (95% CI)	16.0 (14.4, 17.6)	26.6 (19.0, 34.2)	−10.6 (−18.4, −2.8)	0.009
Mean total hospital charges, $USD (95% CI)	44,669 (37,546, 51,792)	44,450 (32,233, 56,667)	219 (−14,155, 14,592)	0.98
Mean total length of stay, days (95% CI)	2.2 (0.8, 3.6)	3.4 (2.2, 4.6)	−1.2 (−3.0, 0.7)	0.22

Abbreviations: CI, confidence interval; *d*, difference; DKA, diabetic ketoacidosis; ED, Emergency Department; eDKA, euglycemic diabetic ketoacidosis; ICU, intensive care unit; USD, United States Dollars.

^a^ *n* = 44 encounters from 38 patients.

^b^ *n* = 15 encounters from 14 patients.

In this analysis of adult ED patients with eDKA treated with a novel eDKA‐specific protocol using an increased rate of dextrose supplementation, compared to patients with eDKA treated with a standard hyperglycemic DKA protocol, we observed a non‐statistically significant reduction in hypoglycemia while on insulin infusion. To our knowledge, this is the first published report describing outcomes with an eDKA‐specific treatment protocol. Cardona et al. [8] described their derivation of a similar eDKA‐specific strategy of fixed‐rate insulin with titratable dextrose‐containing fluids, and highlighted the need for data evaluating safety and efficacy, which our current study helps to provide. As recent guidelines issued by major diabetes associations do not provide specific management recommendations for eDKA due to a paucity of outcome data of treatment strategies [8], our findings can begin to inform recommendations for management of this growingly prevalent condition.

In the post‐intervention cohort treated with greater dextrose supplementation, we suspect three cohort characteristics may contribute to the *non‐statistically significant* trend toward lower incidence of hypoglycemia while on insulin infusion: the post‐intervention group was older, presented to the ED with non‐statistically significantly lower glucose levels, and required a longer duration of insulin infusion than the pre‐intervention cohort. Each of these characteristics could increase the likelihood for hypoglycemia; thus, the effect size to observe a reduction in hypoglycemia may have been weakened. Considering these factors, the observed trend toward lower incidence of hypoglycemia in the post‐intervention cohort could support a protective effect of increased dextrose supplementation. We conducted a multivariable logistic regression analysis of group (pre‐ vs. post‐intervention) as a predictor of hypoglycemia (glucose < 70 mg/dL), statistically controlling for age and presenting glucose. Consistent with bivariate findings, results from multivariable logistic regression analysis showed that being in the post‐intervention group was associated with lower adjusted odds of hypoglycemia, adjusted odds ratio 0.30 (95% CI = 0.03, 2.9), but this association was not statistically significant, *p* = 0.29.

We hypothesize the longer duration of insulin infusion in the post‐intervention group could be related to the higher concentration of dextrose administered, but the retrospective observational nature of our present study is limited to observation of association rather than direct causation. Importantly, iatrogenic hypoglycemia remained a risk even with this dedicated treatment strategy. Since implementation of our eDKA protocol, new guidelines now define eDKA as a blood glucose < 200 mg/dL rather than < 250 mg/dL [1]. In our cohort, despite a higher glucose threshold to diagnose eDKA (< 250 mg/dL), the incidence of iatrogenic hypoglycemia remained relatively high (13.3%). We hypothesize that lowering this glucose definition of eDKA may further increase the incidence of iatrogenic hypoglycemia during insulin therapy, thus heightening the value of an eDKA‐specific treatment protocol with higher dextrose supplementation.

A limitation is all patients were treated in a unique ED‐ICU setting [12], and results may not be generalizable to all settings. The retrospective pre‐post comparison has inherent limitations specific to this approach, and temporal trends and practice variation amongst clinicians may have confounded observed results. The relatively small sample size may limit detection of statistically significant safety and resource utilization outcomes, particularly those with rare occurrences. An automated EHR search was used, and data points might be prone to entry error [13].

In conclusion, an eDKA‐specific treatment protocol using an increased rate of dextrose supplementation was associated with a non‐statistically significant reduction in hypoglycemia while on insulin infusion as compared to a standard hyperglycemic DKA strategy. Iatrogenic hypoglycemia remains a risk during eDKA treatment, even with a dedicated treatment strategy utilizing an increased rate of dextrose supplementation. With the growing prevalence of eDKA, further development and optimization of eDKA‐specific treatment strategies to mitigate the risk of hypoglycemia and promote patient safety are needed.

## Author Contributions

Study concept and design: A.T.C., J.S., and N.L.H.; Acquisition of data: N.L.H.; Analysis and interpretation of data: A.T.C., J.S., J.A.C., and N.L.H.; Drafting of the manuscript: A.T.C., J.S., J.A.C., and N.L.H.; Critical revision of the manuscript for important intellectual content: A.T.C., J.S., L.A., N.H.E., J.A.C., and N.L.H.

## Funding

This work was supported by the University of Michigan Caswell Diabetes Institute Clinical Translational Research Scholars Program.

## Disclosure

Declarations: No large language model (e.g., Rytr or ChatGPT) was used to write any part of the submission.

## Conflicts of Interest

The authors declare no conflicts of interest.

## Data Availability

The datasets used and/or analyzed during the current study are available from the corresponding author on reasonable request.
